# Effectiveness of Epidemic Preventive Policies and Hospital Strategies in Combating COVID-19 Outbreak in Taiwan

**DOI:** 10.3390/ijerph18073456

**Published:** 2021-03-26

**Authors:** Ting Wan Tan, Han Ling Tan, Man Na Chang, Wen Shu Lin, Chih Ming Chang

**Affiliations:** 1 Department of Nursing, Hsinchu MacKay Memorial Hospital, Hsinchu 30071, Taiwan; m617@mmh.org.tw (T.W.T.); m008@mmh.org.tw (M.N.C.); wensha@mmh.org.tw (W.S.L.); 2 Department of Nursing, Yuanpei University of Medical Technology, Hsinchu 30015, Taiwan; 3 National Orthopaedic Centre of Excellence in Research and Learning (NOCERAL), Department of Orthopaedic, Faculty of Medicine, University Malaya, Kuala Lumpur 50603, Malaysia; 4 Department of Healthcare Management, Yuanpei University of Medical Technology, Hsinchu 30015, Taiwan

**Keywords:** epidemic preventive policies, hospital strategies, COVID-19, Taiwan

## Abstract

(1) Background: The implementation of effective control measures in a timely fashion is crucial to control the epidemic outbreak of COVID-19. In this study, we aimed to analyze the control measures implemented during the COVID-19 outbreak, as well as evaluating the responses and outcomes at different phases for epidemic control in Taiwan. (2) Methods: This case study reviewed responses to COVID-19 and the effectiveness of a range of control measures implemented for epidemic control in Taiwan and assessed all laboratory-confirmed cases between 11 January until 20 December 2020, inclusive of these dates. The confirmation of COVID-19 infection was defined as the positive result of a reverse-transcriptase–polymerase-chain-reaction test taken from a nasopharyngeal swab. Test results were reported by the Taiwan Centers for Disease Control. The incidence rate, mortality rate, and testing rate were compiled, and the risk ratio was provided to gain insights into the effectiveness of prevention measures. (3) Results and Discussion: This study presents retrospective data on the COVID-19 incidence rate in Taiwan, combined with the vital preventive control measures, in a timeline of the early stage of the epidemic that occurred in Taiwan. The implementation of multiple strategy control measures and the assistance of technologies to control the COVID-19 epidemic in Taiwan led to a relatively slower trend in the outbreak compared to the neighboring countries. In Taiwan, 766 confirmed patients were included, comprised of 88.1% imported cases and 7.2% local transmission cases, within the studied period. The incidence rate of COVID-19 in Taiwan during the studied period was 32 per million people, with a mortality rate of 0.3 per million people. Our analysis showed a significantly raised incidence risk ratio in the countries of interest in comparison to Taiwan during the study period; in the range of 1.9 to 947.5. The outbreak was brought under control through epidemic policies and hospital strategies implemented by the Taiwan Government. (4) Conclusion: Taiwan’s preventive strategies resulted in a drastically lower risk for Taiwan nationals of contracting COVID-19 when new pharmaceutical drug or vaccines were not yet available. The preventive strategies employed by Taiwan could serve as a guide and reference for future epidemic control strategies.

## 1. Introduction

The coronavirus disease 2019 (COVID-19) infection was first identified in Wuhan, China at the end of 2019 and has rapidly spread across the world [1]. A broad spectrum of symptoms has been reported, from asymptomatic and mild symptoms to severe acute respiratory syndrome and respiratory failure [1,2].

The incidence rate of COVID-19 in China was on the rise since December 2019, rapidly spreading to other countries and continents [1]. The World Health Organization (WHO) declared the outbreak a Public Health Emergency of International Concern on 30 January 2020. The COVID-19 outbreak impacted not only the health sector, but affected the global economy equally if not more, as most companies and significant manufacturers were restricted or temporarily shut down, and the countries most touched by the epidemic instituted travel bans to control the spread. The outbreak has shaken the global economy, powerfully upsetting every primary industry, from food, fashion, and entertainment to automobiles and technologies [3]. Taiwan is an island 130 km off China’s coast, which had 404,000 Taiwanese citizens commuting to and from China in 2019 [4,5]. During the Chinese Lunar New Year festival season in late January, approximately 1.41 million inbound travelers came into Taiwan, among which 230,000 were from China [6]. With the high traffic moving across Taiwan’s border daily, the risk of importing the virus rose proportionately, and was considered the highest risk of virus import from abroad [5]. However, since early January, the Taiwan Centers for Disease Control (TCDC) anticipated this situation and took measures early, developing policies to manage the epidemic crisis by identifying, isolating, and halting further transmissions in the early stage to minimize the spread of the virus and overcome the crisis of the COVID-19 pandemic.

In this study, we aimed to analyze the results of the COVID-19 epidemic prevention strategies in Taiwan. Policies and strategies were verified by analyzing daily confirmed cases and deaths based on the open information announcements from the TCDC government website.

## 2. Materials and Methods

### 2.1. Study Design

The research team conducted a case study. Data, policies, and strategies were collected, along with descriptive analyses, to measure the responses against Taiwan’s COVID-19 outbreak.

### 2.2. Data Sources

Basic data were collected, including demographic information, clinical symptoms, policy plan, and reported data from the electronic records of the TCDC and WHO websites, respectively, from 11 January to 20 December 2020. Accumulative daily new confirmed cases, COVID-19 reverse-transcriptase–polymerase-chain-reaction (RT-PCR) tests, and fatalities were extracted.

### 2.3. Study Population

The study included patients with laboratory-confirmed COVID-19 infections. A confirmed case of COVID-19 was defined as a positive RT-PCR test, collected with a nasopharyngeal swab. The data compiled included demographic information, transmission classification and clinical characteristics, and clinical outcome, as well as the epidemiology of COVID-19. The measurement of outcomes was determined based on the incidence rate and mortality rate. The analysis was done by comparing the testing rate and incidence rate of Taiwan and neighboring countries, and the risk ratio was calculated.

### 2.4. Statistical Analysis

Categorical variables were summarized as frequencies, and percentages (%) were used to describe the available data.

## 3. Results

### 3.1. Epidemiology

#### 3.1.1. Demographic and Clinical Characteristics of Confirmed Cases

The first confirmed case was on January 21. A cumulative total of 766 cases were confirmed and a total of 119,405 RT-PCR tests were conducted up to 20 December 2020. Of these 766 confirmed cases, 88.1% were imported cases from overseas, 7.2% were local transmission cases, and 4.7% were reported from the Navy Dunmu Fleet cluster. Epidemiologically, there was a near-equal distribution in terms of gender involvement; male 46.5% and female 53.5%. There was an age distribution of 10.9% in the age group of 60 years and older, versus 89.1% in individuals 59 years and younger.

Symptomatically, the top five clinical features presented were flu symptoms (running nose, nasal congestion, sore throat, slight dyspnea) (39.1%), followed by cough (18.8%), fever/chills (17.4%), anosmia (5.4%), gastrointestinal symptoms (diarrhea, vomiting, stomachache) (5.4%), and asymptomatic patients (15.9%), as shown in Table 1.

#### 3.1.2. Clinical Outcomes

Among 766 confirmed cases, 135 (17.6%) received a diagnosis of pneumonia; 99 (73.3%) reported non-severe pneumonia, and 36 (26.7%) reported severe COVID-19 pneumonia or acute respiratory distress syndrome. Of patients with severe disease, 24 (5.4%) underwent invasive mechanical ventilation, and three (0.7%) underwent extracorporeal membrane oxygenation treatment.

#### 3.1.3. Fatality

Out of the cumulative total of seven COVID-19 death cases reported during hospitalization, 85.7% had at least one coexisting underlying chronic illness, such as hypertension, diabetes, coronary heart disease, chronic renal disease, hepatitis B, cancer, hyperlipidemia, and overweight. Higher mortality rates were reported in males, at 86%, and lower rates were reported in females, at 14%. Five fatality cases were under the age of 65 (71.4%) and two fatality cases were above the age of 65 (28.6%).

#### 3.1.4. COVID-19 Incidence Rate, Mortality Rate, and Testing Rate of Taiwan and Neighboring Countries with Risk Ratios of Neighboring Countries

**Figure 1:**
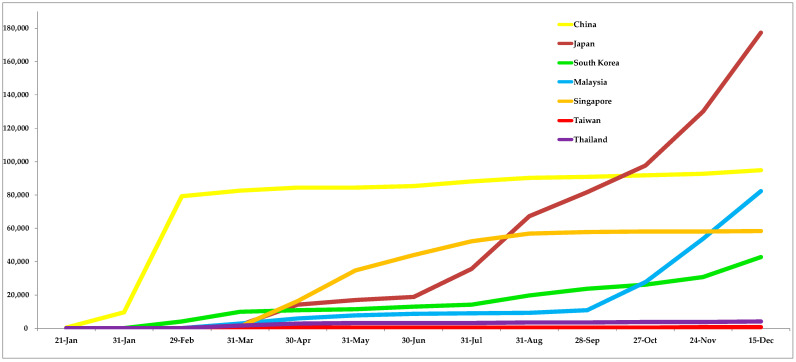
Cumulative number of confirmed COVID-19 case in Taiwan and neighboring countries [7].

The cumulative COVID-19 confirmed cases in Taiwan and neighboring countries as of 20 December 2020 are shown in Figure 1. The incidence rate of COVID-19 in Taiwan was 32 per million citizens, and the mortality rate was reported to be 0.3 per million citizens. RT-PCR test rates were reported to be 5009 per million citizens, compared to neighboring countries close to Taiwan. In terms of the risk ratio of COVID-19 compared to Taiwan, Chinese nationals were 1.9 times as likely to get COVID-19 as compared to Taiwanese nationals, whereas Japan was found to have 50.3, Malaysia had 947.5, Singapore had 311.3, South Korea had 32.0, and Thailand had 2.6 times the risk of contracting COVID-19 compared to Taiwan, as shown in Table 2.

The WHO reported in a total of 218 countries affected, with a total of 75,129,306 confirmed cases, and 1,680,794 fatalities globally up to December 20, 2020 [7,9]. Only imported COVID-19 confirmed cases were reported and there were zero locally transmitted cases in Taiwan in the last 252 days of the period (April 12 to December 20, 2020). As of December 20, 2020, a cumulative total of 766 confirmed cases in Taiwan had been reported, with seven fatality cases [10], 627 recovered patients, and 132 patients still under hospital care [10].

### 3.2. Summary of the COVID-19 Epidemic Status in Taiwan

The first confirmed Taiwanese case of COVID-19 was reported on 21 January 2020—a Taiwanese person who worked in Wuhan, China was detected at Taiwan Taoyuan International airport. The majority of confirmed cases reported were imported cases—people who traveled or worked in Wuhan, China, during the Chinese Lunar New Year. There was higher traffic across the Taiwan border in late January and early February, with a peak of 17 confirmed cases (15 imported and two locally transmitted cases) reported from 21 January to 8 February 2020.

The majority of local transmissions were the close contacts of family members, relatives of the positive individuals returning from China to celebrate Chinese Lunar New Year, which lasted from 9 February until 3 March 2020—a total of 25 confirmed cases (21 locally transmitted and four imported cases).

Case incidence peaked in mid-March, with Taiwan citizens who contracted the infection overseas evident by their histories of travelling to other countries during the Chinese Lunar New Year holiday, as well as Taiwanese students and citizens returning from abroad, with a total of 280 confirmed cases (257 imported from overseas and 23 locally transmitted cases).

After the implementation of travel restrictions and stricter border control measures, the incidence rate decreased in early April. However, the case incidence peaked again in mid-April, with a cluster of infections identified among the naval crew members of the Dunmu fleet, as the naval fleet returned to Taiwan from overseas training exercises from 5 March to 9 April. Subsequently, there was a rapid increase in close contact cluster transmission between navy members. In this period there was a total of 118 confirmed cases (73 imported, 36 Navy cluster cases, and nine local transmissions).

**Figure 2:**
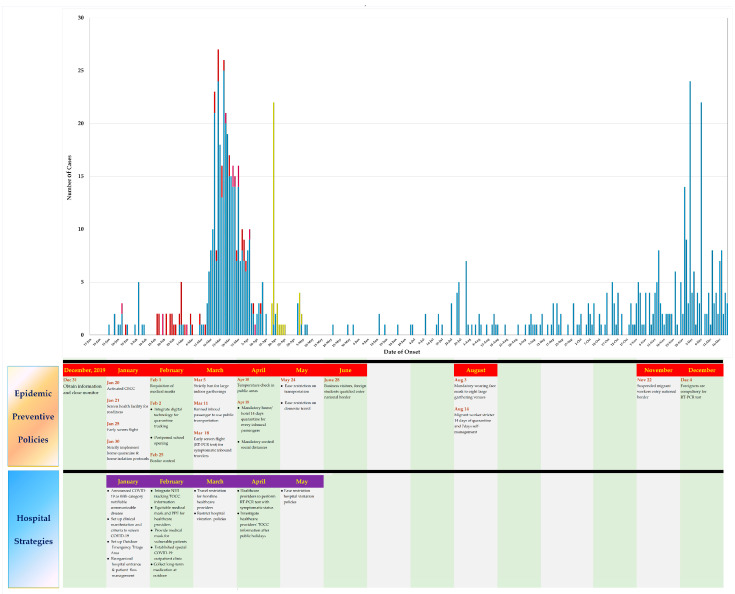
COVID-19 daily confirmed cases reported in Taiwan.

The incidence rate dropped in the month of May, from 8 May to 27 May 2020, with only one imported confirmed case reported. The incidence rate peaked again in the month of June, as foreigner workers with legal residence documents, international students, and approved business visitors started entering Taiwan in early June; a total of 325 imported cases were reported from late May to 20 December 2020. There were spikes in cases that occurred in late November and in late December, with the majority among asymptomatic migrant workers from abroad, who tested positive for COVID-19 at the end of their isolation period in Taiwan, as shown in Figure 2.

### 3.3. Epidemic Preventive Policies and Hospital Strategies Implemented in Taiwan

Taiwan successfully implemented epidemic preventive policies and hospital strategies to control the COVID-19 pandemic; a summary of the essential measures is presented in Table 3.

#### 3.3.1. Early Response and Centralized Central Epidemic Command Center (CECC) Professionalism

The TCDC detected COVID-19 epidemiologic information in the early stages, while neighboring countries reported ongoing community transmission and a severe special infectious pneumonia outbreak, and received information from the inter-ministerial meeting on 31 December 2019. Two experts visited Wuhan, China, to obtain information in early January. The TCDC activated the Central Epidemic Command Center (CECC) and announced COVID-19 as a fifth-category communicable disease and closely monitored the status of the epidemic.

The centralized command system of the CECC assessed public and private health sector readiness. It established clinical manifestations and criteria for COVID-19, screened and reorganized all hospital entrance points, ensuring patient flow management, and screened medical resources for disease control actions in late January.

#### 3.3.2. Knowledge and Awareness

The public was updated with the latest information and the latest preventive precautions by the TCDC. Online platforms, media, and posters were used as a part of anti-coronavirus efforts to raise public awareness of the COVID-19 pandemic.

#### 3.3.3. Precautionary Measures

Taiwan had experienced SARS in 2002, which had established a culture of face mask use by the public. The TCDC sequestrated medical facial masks for the public and controlled the export of masks to ensure a steady supply of masks and prevent sudden shortages due to panic buying and hoarding of masks by the public, and had a secure supply for citizens from early February. Inventory checking of medical devices and equipment for healthcare sectors was undertaken to efficiently allocate medical supplies and devices in healthcare facilities, which reduced the possibility of shortages during the epidemic. They mandating the wearing of face masks in eight large gathering venues, including healthcare settings, public transportation, markets, schools, sports and exhibition venues, religious venues, entertainment venues, and large-scale events; violations of the related rules were subject to cumulative penalties in early August.

All primary, secondary schools, and colleges postponed school opening in the first spring semester for two weeks after the Chinese Lunar New Year holiday. In four thousand school campuses and facilities, sanitization measures were implemented, and the rearrangement of the seats was undertaken to ensure a distance of at least 1.8 m between desks before school opening. Maintaining the classrooms’ airflow was accomplished by opening all the windows and implementing social distancing protocols to prevent students from assembling in crowded areas, such as playgrounds and halls. Students were educated on the measures to prevent the spread of virus infection, such as adherence to temperature checking, preparing their own masks, and handwashing with soap regularly.

#### 3.3.4. Big Data Analytic Tracking System for Case Identification and Large-Scale Surveillance

Inbound passengers were instructed to complete the “Novel Coronavirus Health Declaration Form” online. This contributed to the use of big data technologies to map neighboring populations to conduct contact tracing. Taiwan’s national health insurance databases were integrated with immigration and customs databases, which assisted in tracking high-risk individuals as identified using the COVID risk assessment tool. The big data technologies were integrated with digital technology for quarantine tracking of individuals through the national health insurance (NHI) MediCloud System. Big data technologies were also utilized in the implementation of a rationing system to equally distribute resources for the public to purchase using unique personal national health insurance (NHI) cards.

Inbound passengers were instructed to and required to undergo home quarantine or home isolation for 14 days upon arrival. Passengers who were under quarantine were given government-issued mobile phones and monitored with calls and visits. If a person under home quarantine or isolation had developed symptoms, health agencies would arrange for medical treatment as needed.

#### 3.3.5. Border Control and Border Quarantine Measures

Travel bans and restrictions were in place from late January, initially focusing on passengers moving between China and Taiwan. Passengers who arrived from affected area were screened and conveyed for further investigation. In early March, travel advice was given in the form of a global travel warning that Taiwanese citizens should avoid all non-essential travel abroad to all countries. During mid-March, airport screening of passengers inbound to Taiwan was implemented; for all inbound travelers who exhibited symptoms of COVID-19, an RT-PCR screening test had to be done in the airport. During April, the government extended restrictions on direct cross-strait flights and implemented a ban on passenger transits through airports. Inbound travelers from overseas were advised to notify health officials before returning to the national border. All inbound passengers were banned from taking public transportation, such as buses, subway, high-speed rail, trains, and ferries. Passengers were only allowed to use compulsory quarantine taxis. All inbound passengers from overseas had to undergo quarantine at home or compulsory quarantine hotels or facilities for 14 days. The local city council set up a COVID-19 consultation center and support services to perform daily follow-up calls by community nurses. These support services included transport arrangements, medical care arrangements, household services, settlement planning, meal delivery, garbage collection, and consultation services.

#### 3.3.6. Social Distancing Measures

During early March, mandatory rules were introduced for social distancing measures due to COVID-19; to keep a distance of at least 1.5 m indoors and 1 m outdoors. Regulations were implemented for the prohibition of large-scale gatherings, including indoor and crowd gatherings. The annual religious festivals and celebrations were postponed and rescheduled to curb the spread of the virus. Individuals with respiratory symptoms were instructed to perform home quarantine and seek medical assistance immediately.

#### 3.3.7. Healthcare Facilities Provisions and Medical Team Preparations

The Communicable Disease Control Medical Network (CDCMN) identified six regions (Taipei, north, central, south, Kao-Ping, east) in Taiwan and designated respective healthcare centers for COVID-19 patients in each region to provide isolation facilities and massive training courses. Responding hospitals would serve as backups for medical resources and workforces to be responsible for isolating patients with emerging infectious diseases (EID).

Travel restrictions were implemented for frontline healthcare providers and healthcare providers underwent RT-PCR testing if they reported a symptomatic status. Healthcare settings established COVID-19 hospital visiting policies, including a limited number of visitors and visiting hours allowed on an ordinary ward. All visitors were screened with a COVID-19 risk assessment and monitoring of body temperature before entry to hospitals. For all visitors and accompanying family, it was mandatory to put on a face mask and to keep strict records for tracking purposes, in order to conduct contact tracing if needed.

#### 3.3.8. Restrictions for Foreign Migrant Workers

The gradual easing of restrictions on hospital visitation policies in specific circumstances, as well as of restrictions for public transportation and domestic travel were planned in mid-May. However, imported cases were reported from international students, business visitors, and migrant workers from June until December. Foreign migrant workers were required to provide pre-entry COVID-19 test reports before entry to the national border in mid-June. Entry restrictions and a temporary suspension of migrant foreign worker entries were implemented in mid-November. It was compulsory for them to undergo 14 days of quarantine in certain facilities, followed by a 7-day self-health management period and a compulsory COVID-19 test in early December.

## 4. Discussion

EIDs are emerging infective diseases that are not yet well known by the authorities, and it is crucial to integrate multiple modalities both within countries and abroad, in combination with the latest research and investigations, to obtain knowledge regarding epidemiological information and to develop policies for each stage of the epidemic. Rapid response action aims to activate control measures in different epidemic stages in managing a crisis to prevent further outbreaks [11] and to establish the differential clinical manifestations with criteria that can be used by healthcare experts to screen for COVID-19, using preventive medicine to assist healthcare workers in preparing to confront the emerging threat of COVID-19 [12].

The latest information was provided and updated via various approaches, with an online platform in different languages, as well as the use of sign language regarding COVID-19 cases. This study revealed that the delivery of timely, accurate, and transparent information regarding the evolving epidemic and notifying the public about travel warning levels across the world, promoting disease awareness, reassuring the public, proactively clarifying information when necessary, and avoiding rumors or misunderstandings to prevent panic are vital roles for the authorities [11]. It is crucial for the public to self-educate, cooperate, minimize panic, and prevent social stigma, which are similar suggestions given by Hellewell [13].

Tracking systems using big data technologies enable authorities to map neighboring populations, conduct contact tracing to improve infection control, collaborate with telecom companies, and utilize electronic security monitoring systems to locate the mobile phones of individuals under surveillance remotely. If a person is away from the designated quarantine area, the phone signal will move to the nearest cell towers, allowing civil affairs bureau workers to notify and warn individuals with notifications via SMS, and instruct them to return. Chen et al. [14] concluded that this method is an efficient method to track down infected persons, map cases, identify the sources of infection, and control the exposure of infected hosts and the spread of COVID-19. Cheng et al. [15] suggested that contact tracing analysis in the early stages provides a useful strategy to minimize transmission and ensure shorter periods of infection. Hellewell et al. [13] identified in their modeling study that isolation and contact tracing would decrease transmission, which has a large effect on the possibility of achieving control of the virus within 3 months. Border control measures were carried out to restrict all non-essential travel abroad, along with airport screening for all inbound passengers, and for those who exhibited symptoms of COVID-19, RT-PCR testing was performed when necessary, which enabled the early detection of imported cases and assisted in preventing widespread community transmission. As reported in several studies, COVID-19 has a short infectious period, which generally occurrs with a median of 4 to 5 days after initial symptom onset [15], with a gradual decrease in viral load after ten days [16], which strongly suggests that a high transmissibility of COVID-19 occurs before the onset of symptoms. Other responses related to suspect infected inbound passengers, included the ban on the use of public transportation and compulsory quarantine taxis to prevent close contact with local residents and reduce the chances of community transmission. Every inbound passenger was required to undergo quarantine and strictly instructed to perform self-management with 14 days of home quarantine or compulsory hotel quarantine, remaining separate from vulnerable relatives, thus decreasing the interval between symptom onset and isolation, preventing local clusters and the wide spread of COVID-19. Wells et al. [17] demonstrated that the implementation of border control measures such as airport screening, rapid contact tracing, and border quarantine for asymptomatic inbound passengers during the incubation period were effective ways to limit transmission of the pandemic in the community.

The mandatory social distancing measures included banning crowds and mass gatherings and instructing the public to wear a face mask to protect themselves and others. Temperature checks were implemented in public areas. People who had traveled to crowded regions and who presented with respiratory symptoms were required to perform home quarantine and seek medical assistance immediately to decrease the proportion of community transmission. Cheng et al. [15] suggested that aggressive social distancing was an efficient way to reduce community mobility, along with proactive contact tracing, and that it could block the COVID-19 transmission chain and keep probable patients away from high-risk vulnerable populations [18].

Hospital administrations are vital in order to minimize the exposure risk of frontline workers and ensure frontline healthcare workers are aware of the latest information and updates. Regular monitoring and training are essential to maintain staff competencies, improve compliance with hospital protocols, protect vulnerable patients, and achieve an ideal environment to prevent the spread of diseases. With the aim of reducing the peak in healthcare demand, the CDCMN was activated to centralize patients with severe highly infectious diseases and to designate appropriate supporting healthcare centers and provide better care and treatment. If an outbreak expands further, the CDCMN will activate and appoint requisition healthcare facilities at different levels to prioritize patients with emergency or outbreak situations. The preparedness of the hospital network and healthcare system is crucial to protect frontline healthcare workers and the public from EID threats by reducing nosocomial transmission within hospitals [12].

Most confirmed cases in Taiwan were aged between their 20s and 40s, with a higher percentage imported from overseas. Most confirmed cases were reported to be asymptomatic or non-severe symptom carriers. The low incidence of local transmission was partially due to the implementation of digital tracing policies for suspected and RT-PCR-tested individuals, and the measures taken against violators after warnings. Taiwan’s epidemiology supports the suggestion by Klinkenberg et al. [19] that quarantine interventions, symptom monitoring strategies, and contact tracing are vital to control EIDs that are not yet well-known. It is favorable to apply an iterative process of contact tracing at the beginning of an epidemic, and it is efficient to trace asymptomatic and unidentified infected individuals to control with suspected exposure to EID [20].

We believe the implementation of crucial policies in a timely fashion in accordance with the progress of the epidemic is the key in controlling local transmission. Well-informed health experts were able to identify the timeline, phases, and severity of local transmissions under each circumstance and implement policies ahead of time, in order to halt further transmissions. Our analyses are consistent with the suggestion by Peak et al. [20] that non-pharmaceutical interventions are necessary to control an emerging pathogen’s outbreak when the pharmaceutical medications or vaccines are still in development.

The public was informed in the case of early suspected symptoms and signs of infection to approach the nearest healthcare center for medical assistance. Taiwan’s national health insurance provided equal medical care and coverage for residences, lowering the fear of a financial burden for the public [21]. With the combination of all the epidemic preventive policies and hospital strategies in Taiwan, our analysis showed that citizens of other neighboring countries were 1.9 to 947.5 times more likely to contract COVID-19 than those of Taiwan during the COVID-19 outbreak.

Despite drastic differences in incidence rates between Taiwan and China, they have many similarities in terms of COVID-19 epidemiology and control measures, which were addressed by Wu et al. [22]. Epidemiologically, confirmed cases were more common among young adults and middle-aged people. Taiwan and China learned from their disastrous experiences during the SARS outbreak in the year 2002. Both have established prompt and improved public health response mechanisms in responding to the COVID-19 epidemic, compared to SARS [22]. A few similar policies (border quarantine measures, border control, and community surveillance) were employed by both Taiwan and China to fight against COVID-19. However, Chinese authorities acted more aggressively to lock down the Wuhan market and terminate all business trades. Several affected cities were also placed in lockdown and quarantine stations were set up to check the entrances and exits to the cities, screen residents’ houses, and force ill patients to be isolated. There were established specialized hospitals at the provincial level to relieve the overstressed healthcare system. Evidently, the spread of COVID-19 in China was effectively and steadily controlled soon after implementing these policies by breaking the transmission. On the other hand, the Taiwan government recognized, anticipated, and responded much earlier, before any imported cases. Subsequently, the early implemented strategies were shown to reduce the overall impact of the COVID-19 outbreak.

Japan and Thailand had a relatively slower responses in implementing the immigration restrictions and introduced weaker measures to control the entry of the foreigners across the national borders, resulting in numerous infection clusters occurring simultaneously across the country [23,24]. Both countries also implemented several policies such as social gathering policies, temporary closures of business and facilities, maintaining social distancing, screening, and suspending incoming flights to control the transmission and ease the outbreak [23,24]. However, late responses might not be as effective as early implementation, and once the epidemic occurred at the community level, the subsequent control may be more challenging, as demonstrated by Japan and Thailand’s situation in epidemic control [24,25]. In contrast, Taiwan had an immediate response, deciding to screen all inbound passengers and restricting travel for Taiwanese citizens and those with resident status from February onwards.

Malaysia’s government faced political issues during the COVID-19 pandemic, which caused a slight delay in Malaysia’s response to the pandemic [26]. In contrast, Taiwan’s ruling party was elected in early January 2020, and retained its legislative authority over the CECC and TCDC, which obtained full political support. A stable and unanimous government plays a vital role in public health policymaking [27], enabling the government to handle the crisis with a society that is willing to collaborate and attempt to eradicate the epidemic.

Taiwan and Singapore successfully implemented several similar public health interventions to control the pandemic using a broad-based and sensitive surveillance system, rapid and effective contact tracing, and a low threshold for enforced quarantine during the earlier stage of epidemic [28,29]. However, Singapore had a delayed response towards the outbreak and experienced delays in assessing and addressing the infection risks, resulting from its dense population and lack of focus on foreign worker dormitories and communities [29]. By contrast, Taiwan’s foreign migrant workers led the massive number of confirmed imported COVID-19 cases reported in June to December. Taiwan mandated a compulsory 14 days of quarantine in a specific well-ventilated room, in facilities with one room per person, followed by a 7-day self-health management period, as well as requiring people to undergo COVID-19 testing both during quarantine and after quarantine period [30].

South Korea reported several clusters of local transmissions, associated mainly with entertainment venues and religious gatherings and events. The late implementation of massive COVID-19 epidemic measures, such as isolation, strict social gatherings, usage of face masks, limiting social distancing, surveillance, and heightened border controls, might not be as effective as early implementation [31]. South Korea then adopted high-volume comprehensive testing, and localized strong social distancing measures in high transmission areas, which were vital factors in South Korea’s successful outbreak control [31]. By contrast, Taiwan took measures to immediately trace infection sources once a confirmed case was reported. Taiwan implemented strict quarantine measures for all inbound citizens, travelers, foreign business visitors, international students, and migrant workers from March onwards, mandating a required 14-day quarantine period on arrival, followed by a seven-day self-management period from July. In addition, the government’s capacity was strengthened by Taiwan’s robust information technology, which was interlinked with government databases, enabling them to conduct robust contact tracing and tracking. As the transmissions had yet to reach the community level, massive testing would not have been fruitful and cost-effective. Currently, tests are only performed for incoming passengers with COVID-19 related symptoms in Taiwan, as supported by several experts and studies [32,33] stating that mass testing for the general population is inappropriate and should be avoided, resulting in less accurate and misleading test results. The government would face the risk of false reassurance [33], discussed in a study by Ferrari and De Angelis, who argue that potential false-negative RT-PCR test results might occur in low viral cases, as almost 50% of viral transmission occurs within three days before the occurrence of symptoms, which is the best time to perform test [32]. Ideally, every citizen would be tested. However, this is impossible due to material costs and personnel availability limitations. With current restrictions, among high-risk populations, testing should be done based on priority, such as people suspected of being infected, and anyone with close contact with COVID-19 cases, as well as employees in high-density workplaces [32].

Taiwan has gradually eased restrictions on hospital visitation policies in specific circumstances and gradually eased public transportation and domestic travel plans, whereas zero local cases were reported in the last 252 days. In Taiwan, no signs of an emerging COVID-19 wave were noted after the easing of restriction at the end of May 2020. It is noteworthy that the case of South Korea has shown that aggressive emergency measures resulted in gradual slowing down the curve of transmissions. However, a premature termination in border control and quarantine measures led to an earlier rebound of the outbreak [34].

In preparation for the upcoming second COVID-19 wave threats, there is room for improvement in Taiwan and the CECC, with attempts to anticipate the signs of the second wave through early recognition, protocols for the activation of emergency management structures, and bold policy implementations through updated and well-communicated management processes. Big data and the advancement in technologies allow authorities to be well informed regarding the latest updates and assist in obtaining more effective monitoring systems for compliance monitoring and health monitoring. Public responses to an outbreak are strongly affected by education, and it is crucial to avoid panic and fear at the community level. Hence, an easily accessible platform and regular media updates served as the cornerstone of community education. It is recognized that both the government and the public played vital roles in reducing the outbreak’s impact. Hospital strategies are vital to ensuring the safety of frontline workers while providing care to infected patients, in the form of strict adherence to infection prevention guidelines and control in the healthcare setting. Regular surveillance, assessment, and evaluations are to be conducted in order to ensure the system’s awareness and readiness to anticipate any future outbreaks. For the upcoming winter flu season, in conjunction with COVID-19 in Taiwan, the CECC has strongly encouraged every citizen to obtain an annual influenza vaccine and has offered free influenza to healthcare personnel and high-risk populations. All these measures aim to reduce severe complications requiring hospitalization and deaths to alleviate the healthcare burden of COVID-19, and similar suggestions are given in the study by Grech et al. [35].

The COVID-19 pandemic is a global phenomenon, and countries’ responses are local [36], and specific responses against COVID-19 depend on the political culture, governance system, policy capacity, healthcare system, and socioeconomic status of different countries [28]. Most importantly, citizens’ culture, habits, behavior, and sanitation are related to the main policy measures to be undertaken as part of their unique response mechanisms to cope with pandemic events [24]. Setting up acceptable pandemic interventions is vital, in order for citizens to be willing to collaborate with public health regulation, and these should be updated to ensure that pandemic control measures balance the demands to protect citizens’ rights and liberties [37].

In the future, improvements in and extensions of surveillance measures, cooperation, coordination, and communication at the international level, as well as incorporating more technological advances to assist in the control of outbreaks, could have benefits that are not only limited to one nation, but to the whole continent, which could introduce a new era of preventive medicine.

## 5. Limitations

Concerning the limitations of this study, some cases had incomplete documentation of clinical characteristics, as there were variations in the structure of the official electronic databases for data extraction. No imputations were made for some missing data, due to the fact that data were not derived from a random selection, but rather were extracted from the TCDC and WHO websites. Some of the control measures introduced in this study would not be able to be associated with the resulting improvements due to the lack of investigation of the cause-and-effect relationship between variables. We emphasize that future studies with larger sample sizes and prospective study designs are warranted to further explore the inhibition strategies and policies for severe events in COVID-19, prevent the spread of the epidemic, and to anticipate any future outbreaks.

## 6. Conclusions

The impact of the COVID-19 outbreak is enormous, with effects both at the national level and internationally. Many countries have adopted similar public health interventions. However, Taiwan has taken one step ahead to adopt epidemic preventive policies and hospital strategies designed to avert the effects of the pandemic. These policies and the timing of their implementation have made a difference. Taiwan has shown a successful response in preventing the COVID-19 outbreak to date (December 2020) and potentially reducing the outbreak’s impact to a minimum. Although epidemic preventive measures are designed to protect the community from COVID-19, there are negative impacts from such measures as well. For instance, home quarantine and isolation measures have negative mental impacts and social wellbeing effects on individuals. The sense of fear, anxiety, distress, and loneliness, as well as uncertainty regarding the pandemic’s progression, poses synergistic negative effects. Border control and travel restrictions are one of the cornerstone preventive measures in the prevention of COVID-19 infection. However, such policies carry a massive impact on the tourism and hospitality sector, and the restriction on the migration of care workers has a direct impact on Taiwan’s long-term care sector. Economic downturns due to restrictions of manufacturers and temporary shutdowns led to a drastic rise in the unemployment rate and caused significant financial pressure for the affected families. Psychologically, the difficulty of making ends meet led to an increase in depression, frustration, and family conflict in households. Every implemented control measure, in spite of its positive intention, has its own negative effects. Therefore, each government should undertake their own unique response mechanism, devising prevention and control strategies to strengthen their response and overcome the emerging pandemic.

## Figures and Tables

**Table 1:** Epidemiology characteristics of confirmed cases of COVID-19; N = 766.

Clinical Characteristics		Frequency	Percentage (%)
Gender	Male	356	46.5
	Female	410	53.5
Age Intervals, y	0~9	5	0.7
	10~19	33	4.3
	20~29	250	32.6
	30~39	166	21.7
	40~49	154	20.1
	50~59	74	9.7
	60~69	62	8.1
	70~79	18	2.3
	80~89	3	0.4
	>90	1	0.1
Transmission Classifications	Local Transmission	55	7.2
	Imported Case	675	88.1
	Navy Dunmu Fleet	36	4.7
Symptoms	Asymptomatic	199	15.9
	Flu Symptoms	490	39.1
	Cough	236	18.8
	Fever (Chills)	218	17.4
	Loss of Smell	68	5.4
	Gastrointestinal Symptoms	41	3.4
Clinical Measures	Hospitalized	132	17.2
	Discharged Alive	627	81.9
	Died	7	0.9

**Table 2:** COVID-19 incidence, mortality, and testing rates of Taiwan and neighboring countries, with risk ratios of neighboring countries.

Countries	Total Covid-19 Confirmed Cases *	Population *	Incidence Rates * (Per Million Population)	Total Deaths *	Mortality Rates * (Per Million Population)	Total Testing *	Testing Rates * (Per Million Population)	Risk Ratio
Malaysia	98,737	32,564,991	30,320	444	14	3,170,140	97,348	947.5
Singapore	58,495	58,72,383	9961	29	5	5,236,487	891,714	311.3
Japan	203,113	126,289,463	1608	2994	24	4,473,256	35,420	50.3
South Korea	52,548	51,290,555	1025	739	14	3,826,570	74,606	32.0
Thailand	5829	69,884,225	83	60	0.9	1,217,873	17,427	2.6
China	86,899	1,439,323,776	60	4634	3	160,000,000	111,163	1.9
Taiwan	766	23,837,521	32	7	0.3	119,405	5009	1.0

* Population data, COVID-19 confirmed cases, deaths, and testing data taken from reported cases and deaths by country, territory, or conveyance [8].

**Table 3:** Epidemic preventive policies and hospital strategies implemented in Taiwan.

Control Measure	Epidemic Preventive Policies	Hospital Strategies (g. Healthcare Facilities, Provisions, and Medical Team Preparations)
Early response and centralized Central Epidemic Command Center (CECC) professionalism	Receiving information from inter-ministerial meeting Obtaining information and closely monitor COVID-19 Activating Central Epidemic Command Center Screening health facility readiness Activating Communicable Disease Control Medical Network	January Announced COVID-19 as a fifth-category notifiable communicable disease Set up clinical manifestations and criteria to screen for COVID-19 Set up outdoor emergency triage area Reorganized hospital entrances and patients Undertook flow management
b. Knowledge and Awareness	Online platform interaction with the public Epidemiology training programs in communities	February Big Data Analytics Tracking System Integrated national health insurance (NHI) tracking of COVID risk assessment information Equitable medical masks and PPE for healthcare providers Medical masks for vulnerable patients Established special COVID-19 outpatient clinic Arranged the collection of long-term medication at outdoor hospital pharmacies
c. Precautionary Measures	Requisitions of medical and surgical masks Banning the export of face masks Allocation of face masks and medical supply Postponing school opening for 2 weeks Sanitizing all school campus environments before school opening Mandatory wearing of face masks in eight gathering venues	March Perfecting the Healthcare System The Communicable Disease Control Medical Network (CDCMN) assigned 6 regions in Taiwan to designated responsible healthcare centers, to be responsible for isolation patients with emerging infectious diseases (EIDs), and to take in priority patients with emergency or outbreak diseases.
d. Big data analytic tracking system for case identification and large-scale surveillance	Integrating digital technology for quarantine tracking	April Infection Prevention and Control Measures for the Healthcare System Travel restrictions for frontline healthcare providers Restriction of hospital visitation policies Advising healthcare providers to perform PCR testing with symptomatic status Investigating healthcare providers’ TOCC information after public holidays
e. Border control and border quarantine measures	Early screening of flights from mainland China Stricter implementation of home quarantine and home isolation protocols Global Travel notice at Level 3: Warning against all non-essential travel overseas Banning symptomatic inbound passengers from using public transportation Early screening (PCR test) for symptomatic inbound travelers in Taiwan airport Mandatory home/hotel 14-day quarantine for all inbound passengers Extending restrictions on direct cross-strait flights and banning passenger transits through airports in Taiwan Inbound travelers from overseas should notify health officials before returning to Taiwan Easing restrictions on transportation Easing restrictions on domestic travel Business visitors and qualified foreign students can enter thr national border	
f. Social distancing measures	Passengers must wear medical masks in public Temperature checks in public areas Mandatory control of social distance for the public Strictly ban and postpone large public indoor gatherings	May Easing restriction for hospital visits under certain circumstances
g. Restriction for foreign migrant workers and business visitors	Migrant workers strictly undergo 14 days of quarantine and 7 days’ self-management Suspension of the entry of migrant foreign workers to the national border COVID-19 tests are compulsory for foreigners	

## Data Availability

Not applicable.
